# A Series of Glomerular Diseases That Developed After COVID-19 Vaccination

**DOI:** 10.7759/cureus.81085

**Published:** 2025-03-24

**Authors:** Yukako Umezawa, Hitoshi Suzuki, Hitomi Hirose, Hisatsugu Takahara, Shigeki Tomita, Yusuke Suzuki

**Affiliations:** 1 Nephrology, Juntendo University Urayasu Hospital, Urayasu, JPN; 2 Pathology, Juntendo University Urayasu Hospital, Urayasu, JPN; 3 Nephrology, Juntendo University Faculty of Medicine, Tokyo, JPN

**Keywords:** covid-19, gross hematuria, iga nephropathy, mrna vaccine, nephrotic syndrome

## Abstract

Background

Although the coronavirus disease 2019 (COVID-19) vaccine has been shown to be effective in preventing severe COVID-19 infection, many vaccine-related adverse events have been reported with the increasing use of COVID-19 vaccines based on messenger RNA (mRNA). Cases of new-onset and relapsing vaccine-related glomerular diseases, including minimal change disease (MCD), antineutrophil cytoplasmic autoantibody (ANCA)-associated vasculitis, immunoglobulin (Ig)G4-related disease, and IgA nephropathy (IgAN), have been reported.

Methods

We present 30 patients who developed glomerular diseases after COVID-19 mRNA vaccination. We evaluated the clinical characteristics, vaccine types, and outcomes of 30 patients whose urinalysis results indicated proteinuria and/or occult blood after COVID-19 mRNA vaccination. For a definitive diagnosis, we performed a renal biopsy and evaluated their histological findings.

Results

Out of 30 patients, 19 (63.3%) were female, and 11 (36.7%) were male. The median age of the patient was 42.5 years (range, 24-66 years). Seventy-three percent of the patients received BioNTech Pfizer vaccines, and 26.7% received Moderna vaccines (mRNA-1273). Gross hematuria was observed in 83.3% of the patients, and 13.3% had nephrotic syndrome. Twenty patients with IgAN were diagnosed by renal biopsy, while the remaining were diagnosed with MCD (n=3), proliferative glomerulonephritis with monoclonal Ig deposits (PGNMID) (n=1), TAFRO syndrome (characterized by thrombocytopenia, anasarca (edema, pleural effusion, and ascites), fever, reticulin fibrosis/renal dysfunction, and organomegaly; n=1), and anti-glomerular basement membrane (GBM) disease (n=1). Four patients who previously underwent treatment for IgAN experienced exacerbation of urinary abnormalities and disease relapse.

Conclusion

In conclusion, various glomerular diseases were newly diagnosed after COVID-19 mRNA vaccination. Although their short-term outcomes seem favorable, some patients developed serious worsening renal function and nephrotic range proteinuria. Patients with vaccine-related glomerular disease should be monitored long-term to predict prognosis in the future.

## Introduction

Coronavirus disease 2019 (COVID-19) has infected more than 600 million people globally in the third year of the pandemic. Safe and effective vaccines against COVID-19 are necessary to end the pandemic. In December 2020, the first two vaccines were approved by the Food and Drug Administration through emergency use authorization in the United States. These vaccines, developed by BioNTech, Pfizer, and Moderna, are based on the messenger RNA (mRNA) vaccine platform. Safety and efficacy trials reported efficacy rates of approximately 95% after two interval vaccinations, with a low incidence of serious adverse events [1]. However, several immune-mediated reactions have been reported with the increasing availability of COVID-19 mRNA vaccines. Myocarditis and pericarditis associated with mRNA vaccines have been reported more frequently than those associated with inactivated vaccines (AstraZeneca) [2]. Paroxysmal nocturnal hematuria (PNH), idiopathic thrombocytopenic purpura (ITP), and thrombosis have also been reported after COVID-19 vaccination [3]. Furthermore, several glomerular diseases have been reported after COVID-19 mRNA vaccination [4]. The previous report considered that vaccines can trigger autoimmunity through mechanisms such as molecular mimicry, bystander activation, anti-idiotypic networks, and epitope spreading [5]. However, the immunomodulatory effects of COVID-19 vaccination remain unclear. In this case series, we report 30 patients who were newly diagnosed with glomerular diseases after COVID-19 mRNA vaccination and provide considerations for post-vaccination surveillance and management. This study aims to identify the clinical features, diagnoses, and outcomes of glomerular diseases that developed or relapsed after COVID-19 mRNA vaccination. Additionally, it seeks to explore potential associations and mechanisms behind these events.

## Materials and methods

Study patients

We recruited patients aged ≥18 with hematuria or proteinuria after COVID-19 vaccination from January 2021 to December 2022 at Juntendo University Urayasu Hospital, Chiba, Japan. All registered patients were Japanese. Firstly, we excluded patients with urinary stones, urinary tract cancer, women in menstrual periods, and urinary tract infections by ultrasonography, computed tomography, and urine culture. We also excluded pregnant patients and patients who could not consent to renal biopsy. Renal biopsies were performed on 26 patients who were not previously diagnosed with glomerular disease. This study was approved by the ethics review board of Juntendo University Facility of Medicine (M19-0223 and E21-0117) and followed the tenets of the Declaration of Helsinki. 

Clinical information

For each patient, the following data were extracted from their clinical records: demographic characteristics, medical history, medications, and detailed information about gross hematuria after vaccination. The type and number of times of vaccine used, details of gross hematuria, onset of symptoms, and medical history were self-reported by each patient. 

Data collection of clinical parameters

We collected clinical data, including serum and urine data, and evaluated the clinical outcomes of all cases. Laboratory data were obtained during the initial visit of the patients to our hospital and the timing of renal biopsy. The estimated glomerular filtration rate (eGFR) was calculated using the following formula: eGFR (ml/min/1.73m^2^)=194×creatine−1.094×age−0.287 (×0.739 for women). Urinary protein excretion was evaluated using the urinary protein/creatinine ratio (UPCR). Microscopic hematuria was defined as the presence of five or more red blood cells (RBCs) in a single field of view magnified 400 times under a microscope. Microscopic hematuria with dysmorphic RBCs on microscopy is specific for glomerular damage. Relapse of glomerulonephritis is defined as worsening of hematuria and/or increased proteinuria.

Kidney biopsy and diagnosis

We used 16- or 18-gauge needles for kidney biopsy. Glomerular diseases were diagnosed with light microscopy (LM), immunofluorescence microscopy (IF), and electron microscopy (EM). Formalin was used as the fixative for LM, and tissue for EM was placed directly in glutaraldehyde. The tissue for IF was directly frozen. Immunoglobulin A nephropathy (IgAN) was diagnosed by dominant or co-dominant IgA mesangial deposit by IF. Minimal change disease (MCD) was diagnosed with the absence of histological abnormalities in the glomeruli by LM and IF. Proliferative glomerulonephritis with monoclonal immunoglobulin deposits (PGNMID) is a spectrum of monoclonal gammopathy of renal significance. PGNMID is characterized by glomerular deposits of monotypic IgG, with a single heavy chain subclass (most commonly IgG3) and light chain restriction (usually κ) [6]. TAFRO syndrome is characterized by thrombocytopenia, anasarca (edema, pleural effusion, and ascites), fever, reticulin fibrosis/renal dysfunction, and organomegaly. Although there is no specific renal histology on TAFRO syndrome, endothelial cell swelling and a double contour of the glomerular basement membrane with mesangiolysis are often recognized by kidney biopsy [7].

## Results

In this case series, we report 30 patients who developed or experienced a relapse of their glomerular disease after COVID-19 mRNA vaccination (Table 1). The majority of patients were female (63.3%), with IgAN being the most common diagnosis. The median age of the patient was 42.5 years (range 24 to 66 years). The vaccines received were from BioNTech Pfizer (73.2%) and Moderna (26.7%). Twenty-six patients had been newly diagnosed with glomerular diseases after vaccination. Macrohematuria was observed in 22 patients (84.6%), and four patients (15.4%) presented nephrotic syndrome. Three patients (11.5%) developed urinary abnormalities after the first vaccination, 19 patients (73.0%) developed urinary abnormalities after the second vaccination, and four patients (15.3%) developed urinary abnormalities after the third vaccination. We performed a renal biopsy for a definitive diagnosis in 26 patients. Twenty of the 26 patients (76.9%) were diagnosed with IgAN. Two out of 26 patients (7.7%) with nephrotic syndrome were diagnosed with MCD. The remaining patients had PGNMID and antiglomerular basement membrane (GBM) disease. The two patients who presented with worsening renal function were diagnosed with TAFRO syndrome and anti-GBM disease.

**Table 1:** Characteristics of patients with newly diagnosed and relapsed glomerulonephritis after COVID-19 vaccine F: female; M: male; sCr: serum creatinine; eGFR: estimated glomerular filtration rate; UPCR: urinary protein creatinine ratio; U-RBC: urinary red blood cells; IgAN: IgA nephropathy; MCD: minimal change disease; PGNMID: proliferative glomerulonephritis with monoclonal immunoglobulin deposits; TAFRO: TAFRO syndrome; GBM: glomerular basement membrane; PSL: prednisolone; Tx: tonsillectomy; RASi: renin-angiotensin-aldosterone system inhibitor; CyA: cyclosporine; CPA: cyclophosphamide; PE: plasma exchange, HD: hemodialysis

S.No.	Age (years)	Sex	Vaccine	Symptom	Fever	Which times of vaccine	Onset	sCr (mg/dl)	eGFR (ml/min /1.73㎡)	Alb (g/dL)	UPCR (g/gCr)	U-RBC (/HPF)	Diagnosis	treatment	Significant eGFR decline
1	29	F	Pfizer	Macrohematuria for 1 day	+	2	6days	0.63	91	4.1	0.2	>100	IgAN	No treatment	None
2	40	F	Pfizer	Macrohematuria for 2 days	+	2	1 day	0.58	91	4.5	0.0	10-19	IgAN	Tx	None
3	39	F	Pfizer	Macrohematuria for 3 days	+	2	1 day	0.99	51	4.2	1.2	>100	IgAN	PSL + Tx	None
4	29	F	Moderna	Macrohematuria for 1 day	+	2	1 day	0.65	88	4.4	0.2	30-49	IgAN	Tx	None
5	46	M	Moderna	Macrohematuria for 1 day	+	2	1 day	0.92	71	4.4	0.8	>100	IgAN	PSL + Tx + RASi	None
6	45	M	Pfizer	Macrohematuria for 5 days	-	2	1 day	0.82	84	3.8	0.0	10-19	IgAN	PSL + Tx + RASi	None
7	48	F	Moderna	Macrohematuria for 3 days	+	2	1 day	0.80	60	3.9	1.0	>100	IgAN	PSL + Tx + RASi	None
8	44	F	Pfizer	Macrohematuria for 3 days	+	2	1 day	0.70	71	4.0	0.7	50-99	IgAN	PSL + Tx	None
9	40	F	Pfizer	Macrohematuria for 3 days	+	2	1 day	0.63	82	4.3	0.0	>100	IgAN	PSL + RASi	None
10	52	F	Pfizer	Macrohematuria for 2 days	+	2	1 day	0.60	81	4.2	0.0	1-4	IgAN	PSL + Tx	None
11	38	F	Pfizer	Macrohematuria for 2 days	+	2	1 day	0.58	92	4.4	0.0	1-4	IgAN	Tx	None
12	25	M	Pfizer	Macrohematuria for 1 day	+	3	1 day	0.86	91	4.1	1.5	>100	IgAN	PSL + Tx + RASi	None
13	50	F	Pfizer	macrohematuria for 2 days	+	2	unknown	0.58	85	4.7	0.2	30-49	IgAN	PSL	None
14	26	F	Pfizer	Macrohematuria for 3 days	+	1	1 day	0.69	84	3.9	0.5	>100	IgAN	PSL + Tx	None
15	35	F	Pfizer	Macrohematuria for 3 days	-	2	Unknown	0.64	85	4.3	0.5	>100	IgAN	PSL + Tx	None
16	29	M	Pfizer	Macrohematuria for 3 days	+	2	Unknown	0.74	103	4.8	0.0	5-9	IgAN	PSL + Tx	None
17	51	F	Moderna	Macrohematuria for 2 days	+	3	1 day	1.06	44	3.8	1.3	>100	IgAN	PSL	None
18	37	M	Pfizer	Macrohematuria for 2 days	+	2, 3	1 day	1.03	67	3.6	0.9	50-99	IgAN	PSL + Tx + RASi	None
19	42	M	Pfizer	Macrohematuria for 2 days	+	3	1 day	0.99	68	4.6	0.0	>100	IgAN	PSL	None
20	53	F	Pfizer	Macrohematuria for 2 days	+	3	1 day	1.21	37	3.8	2.1	20-29	IgAN	PSL + Tx + RASi	None
21	35	F	Moderna	Macrohematuria for 2 days	+	2	1 day	0.81	65	3.9	2.0	50-99	IgAN relapse	No additional treatment	None
22	54	F	Pfizer	Macrohematuria for 2 days	-	1	1 day	1.45	31	3.9	1.8	50-99	IgAN relapse	No additional treatment	None
23	34	F	Pfizer	Increased hematuria and proteinuria	-	2	1 day	0.48	117	3.8	0.4	20-99	IgAN relapse	No additional treatment	None
24	43	M	Moderna	Macrohematuria for 2 days	+	1, 2	2 days	2.24	27	3.9	0.8	10-19	IgAN relapse	No additional treatment	None
25	24	M	Moderna	Edema	+	2	8 days	0.65	126	1.5	16.1	1-4	MCD	PSL	None
26	44	F	Pfizer	Edema	+	2	3 days	0.75	66	1.7	15.3	1-4	MCD	PSL + CyA	None
27	66	M	Pfizer	Edema	-	2	10 days	1.26	45	1.3	7.0	1-4	MCD	PSL + CyA	None
28	50	M	Pfizer	Edema	-	1	About 2 weeks	0.71	92	1.2	17.4	1-4	PGNMID	PSL	None
29	45	M	Moderna	Macrohematuria, edema	-	1	1 day	1.29	49	1.9	0.7	20-29	TAFRO	PSL	None
30	55	F	Pfizer	Macrohematuria, renal impairment	+	2	3 day	7.22	5	2.3	1.6	>100	anti-GBM disease	PSL + CPA + PE	Initiation of HD

Four patients who had previously been diagnosed with IgAN were treated with corticosteroids. All patients were in the non-remission clinical stage defined as a previous report [8] before vaccination. One patient relapsed after the first vaccination, and three patients relapsed after the second vaccination. Three of the four (75%) patients showed macrohematuria, and all showed increased hematuria and/or proteinuria without worsening renal function (Table 2).

**Table 2:** Clinical data before and after vaccination and treatment in IgAN relapse patients F: female; M: male; eGFR: estimated glomerular filtration rate; UPCR: urinary protein/creatinine ratio; U-RBC: urinary red blood cells

		Pre-vaccination			Six months post vaccination		
Patient number as per Table1	Age (years)/sex	eGFR (ml/min/1.73㎡)	UPCR (g/gCr)	U-RBC (/HPF)	eGFR (ml/min/1.73㎡)	Significant eGFR decline	Additional treatment
21	35/F	63	1.1	50-99	78	None	None
22	54/F	31	0.5	20-29	31	None	None
23	34/F	107	0.3	5-9	104	None	None
24	43/M	32	1.7	10-19	30	None	None

## Discussion

Since the first vaccination of COVID-19 worldwide, adverse events have been reported. Vaccines can be associated with acute myocarditis [9] and autoimmune phenomena such as ITP, autoimmune liver diseases, Guillain-Barre syndrome, rheumatoid arthritis, and systemic lupus erythematosus [10]. There have been increasing reports of vaccine-associated de novo or relapsing glomerulonephritis. Klomjit et al. reported that IgAN was the most common glomerular disease associated with the COVID-19 mRNA vaccine [4]. There have also been sporadic reports of antineutrophil cytoplasmic autoantibody (ANCA)-associated vasculitis, anti-GBM nephritis, ANCA-negative vasculitis, idiopathic membranous nephropathy, and IgG4-related disease [11] as COVID-19 vaccine-related diseases [4, 12, 13]. To the best of our knowledge, we are the first to report the onset of PGNMID and TAFRO syndromes after COVID-19 vaccination [14]. The previous report considered the mechanism for COVID-19 vaccine-induced autoimmune disease as molecular mimicry and immune cross-reaction, adjuvants, bystander activation, epitope diffusion, and polyclonal activation of B cells. [5]

In our case series of 30 patients, all patients received the mRNA vaccine. A total of 86.7% were newly diagnosed with glomerular diseases, and 13.3% experienced IgAN relapse after vaccination. Of these newly diagnosed patients, 76.9% were diagnosed with IgAN. All patients were presented with macrohematuria. As in the previous report, the most common glomerular disease after COVID-19 vaccination was IgAN (Table 3) [4, 15-18]. The absolute risk of glomerular disease increased after exposure to the second or third vaccination dose. It is unclear whether COVID-19 mRNA vaccination induces an abnormal immune response that triggers the deposition of nephritogenic IgA-containing immune complexes in the kidneys, or whether the abnormal immune response induced by vaccination only unmasks the presence of previously formed deposits in patients with IgAN. In our cohort, all patients diagnosed with IgAN for the first time after vaccination showed abnormal urinalysis results before vaccination. Therefore, de novo onset through vaccination is unlikely in patients with IgAN.

**Table 3:** Case series of newly diagnosed and relapsed glomerulonephritis after COVID-19 vaccine IgAN: IgA nephropathy; MCD: minimal change disease; MN: membranous nephropathy; AAV: antineutrophil cytoplasmic autoantibody (ANCA)-associated vasculitis; GBM: anti-glomerular basement membrane nephritis; PGNMID: proliferative glomerulonephritis with monoclonal immunoglobulin deposits; TAFRO: TAFRO syndrome; FSGS: focal segmental glomerulosclerosis; MPGN: membranoproliferative glomerulonephritis; TIN: tubulointerstitial nephritis; non-IgA-PGN: non IgA proliferative glomerulonephritis; IgAV: IgA vasculitis

	IgAN	MCD	MN	AAV	GBM	Lupus nephropathy	Others	Case series
Number of cases (newly diagnosed /relapsed)	24 (20/4)	3 (3/0)			1 (1/0)		PGNMID: 1(1/0) TAFRO: 1(1/0)	This study
	12 (5/7)	11 (7/4)	2 (1/1)	3 (3/0)	2 (2/0)	1 (0/1)	IgG4-related nephritis: 1(0/1) Scleroderma renal crisis: 1(1/0)	Li NL, et al. [13]
	5 (4/1)	2 (1/1)	3 (1/2)	1 (1/0)	1 (1/0)		FSGS: 1(1/0)	Klomjit N, et al. [4]
	2 (2/0)	5 (5/0)	3 (3/0)	1 (1/0)		1 (1/0)	MPGN: 1(1/0) FSGS: 1(1/0) TIN: 3(3/0)	Fenoglio R, et al. [14]
	16 (0/16)	4 (0/4)	2 (0/2)	1 (0/1)		1 (0/1)	Non-IgA-PGN: 1(1/0)	Ota Y, et al. [15]
	12 (6/6)	19 (11/8)		4 (4/0)	2 (2/0)	1 (0/1)	IgAV: 2(2/0) Granulomatous vasculitis: 1(1/0)	Wu HHL, et al. [16].

Four patients who experienced relapsed IgAN after vaccination were in the non-remission clinical stage prior to vaccination [8]. At least in our cohort, none of the IgAN patients in clinical remission relapsed after COVID-19 vaccination. Notably, COVID-19 vaccination does not cause a severe clinical outcome of IgAN. In fact, none of the patients with relapsed IgAN after vaccination required additional immune therapy to recover to their baseline proteinuria before vaccination. Therefore, it is likely that COVID-19 mRNA vaccination activates immune responses that trigger a transient exacerbation of hematuria and proteinuria. On the other hand, for almost all newly diagnosed IgAN patients, steroid treatment was performed.

All patients with MCD were treated with steroids and achieved complete remission. Patients with PGNMID and TAFRO syndrome were also treated with steroids and had complete remission. A patient with anti-GBM disease was treated with steroids, cyclophosphamide, and plasma exchange. However, no treatment was effective, and hemodialysis was initiated.

Based on the results of this study, COVID-19 vaccination does not cause persistent renal dysfunction in patients with IgAN and should not be avoided. Though rare, the development of relatively serious diseases, such as MCD, PGNMID, TAFRO syndrome, and anti-GBM nephritis after vaccination, was observed. Indications for vaccination should be considered based on the COVID-19 pandemic.

Our case series has several limitations. First, the sample size was limited, and the long-term data on these patients were insufficient. Additionally, this study lacks a control group and statistical analysis. Second, this study primarily focused only on proteinuria and hematuria and not on all patients with a change in renal function. Third, there is selection bias in identifying cases and limited generalizability due to demographic/geographic constraints. In general, those who experienced macrohematuria underwent a renal biopsy, which resulted in a definitive diagnosis and treatment. Although short-term outcomes seem favorable, long-term follow-up is vital to predict the prognosis in the future.

## Conclusions

The probability of serious COVID-19 vaccine-related adverse effects is rare. However, several cases of COVID-19 mRNA vaccine-related glomerular disease are reported in our cohort. Although their short-term outcomes seem favorable, some patients developed serious worsening renal function and nephrotic range proteinuria. The mechanisms underlying mRNA vaccine-induced kidney disease remain unclear. More studies are needed to elucidate the underlying biological mechanisms and identify the exact causal relationship.
